# Antecedents of Exercise Dependence in Ultra-Endurance Sports: Reduced Basic Need Satisfaction and Avoidance-Motivated Self-Control

**DOI:** 10.3389/fpsyg.2018.01275

**Published:** 2018-07-31

**Authors:** Julia Schüler, Beat Knechtle, Mirko Wegner

**Affiliations:** ^1^Institute of Sport Science, University of Konstanz, Konstanz, Germany; ^2^Institute of Primary Care, University of Zurich, Zürich, Switzerland; ^3^Institute of Sport Science, Humboldt-Universität zu Berlin, Berlin, Germany

**Keywords:** exercise dependence, self-determination theory, need for competence satisfaction, self-control, endurance sports

## Abstract

Initiating and maintaining sports and exercise behavior are usually discussed in terms of strategies for promoting health. In the present study, we analyzed a sample of extreme endurance sport athletes and set out to predict exercise addiction, which is a facet of a sport-related health risk. We therefore draw on self-determination theory (Deci and Ryan, 1985, 2000), according to which low basic psychological need satisfaction can lead to excessive compensatory behavior. We aim to disentangle the effects of need satisfaction in the activity itself (exercising) and outside the activity (work/leisure) on exercise addiction. Furthermore, we propose anxious self-motivation as a mediator and tested whether it links low basic need satisfaction with exercise dependence. A correlational study with 323 multi-triathlon athletes confirmed our hypothesis that need satisfaction in work/leisure (but not in sports) is negatively related to exercise addiction. Furthermore, only need for competence in both domains (sport, work/leisure) is associated with anxious self-motivation. Mediation models showed that low competence satisfaction leads to anxious self-motivation that in turn predicts exercise addiction. The results are discussed critically in terms of their practical and theoretical implications for promoting health through sport and exercise.

## Introduction

Regular exercise is known to have a positive influence on psychological and physiological health. It fosters emotional well-being (Fox et al., 2006), reduces depression and anxiety (Mutrie, 2000; Wegner et al., 2014; Budde and Wegner, 2018) and is associated with a reduced probability of coronary heart disease and with lower mortality (Bouchard et al., 2000; Katzmarzyk, 2006). Thus, it is no surprise that sport and exercise are discussed as health-promoting strategies. However, what is rarely considered in the literature on health-related effects of sports are the negative consequences of sport. One example is when sports and exercise behavior are performed excessively.

The present research has three aims. First, we want to explain exercise addiction as a sport-related health risk by using another psychological concept which has been found to be linked to health and health impairment: the satisfaction of basic psychological needs (Deci and Ryan, 1985, 2000; Kasser and Ryan, 1999; Reis et al., 2000; Ryan et al., 2010; Ng et al., 2011; Vansteenkiste and Ryan, 2013). Our second aim is to disentangle the effects of basic need satisfaction by examining the overall score as well as the three sub-scores (autonomy, competence, relatedness) and by considering need satisfaction in the exercise activity itself as well as outside the activity (work/leisure). Our third aim is to discuss the role of avoidance-oriented forms of self-control as an explanation for the link between psychological need thwarting and exercise dependence. Anxious self-motivation (Kuhl, 2000), which is such an avoidance-motivated facet of self-control, could serve as a mechanism that links low basic psychological need satisfaction (e.g., need for competence satisfaction) with exercise dependence. Athletes who experience low need satisfaction in other life domains and are anxiously self-motivated may tend to participate in excessive sport because this life domain may promise controllable satisfaction of different basic psychological needs like competence or autonomy.

### Exercise Dependence

Exercise dependence is defined as “a craving for leisure-time physical activity, resulting in uncontrollable excessive exercise behavior that manifests in physiological (e.g., tolerance/withdrawal) and/or psychological (e.g., anxiety, depression) symptoms” (Hausenblas and Downs, 2002, p. 90). Referring to criteria for substance abuse defined in the Diagnostic and Statistical Manual of Mental Disorder (DSM– IV; American Psychiatric Association, 2000), Griffith (1996, 2002) has suggested six components that contribute to behavior addiction such as exercise dependence. These components are *salience* (exercising is ever-present in a person’s life), *tolerance* (the person has to increase the bouts of exercise in order to achieve its positive effects again), *regulation of mood* (use of exercising as a strategy for regulating emotions), *symptoms of withdrawal* (e.g., irritability, a bad mood), *conflicts* with other persons (e.g., demands of family, friends), with other activities (e.g., less time for job) and within the person (conflicts between the impulse to exercise and the wish to free oneself from the dependence), and *relapse* (quick return to the same pattern of behavior and intensity even after remaining abstinent for many years). Exercise dependence is often used interchangeably with the term exercise addiction (Szabo et al., 2018).

Exercise dependence has been found to be associated with low self-esteem, a high need for achievement (Cohen, 1995; Cook, 1996; Szabo et al., 2018), weight concerns (Davis, 1990), interpersonal problems (Rudy and Estok, 1983), social dependency (Smith et al., 1998), high negative affect after missing a workout, and the continuation of exercising despite injuries (Anshel, 1991). Because exercise dependence is characterized by an increased tolerance to physical stress and by excessive exercise behavior, it can also lead to severe physical damage such as reversible, or even irreversible, health impairment and mortality (Cumella, 2005). In summary, excessive exercise has serious detrimental physical and psychological consequences (Hall et al., 2007). It is therefore extremely important to analyze its antecedents.

Our focus in the present research is on psychological antecedents of exercise addiction as compensation of psychological need thwarting in other life domains. We assume that ultra-endurance athletes adhere to an excessive sport program because basic psychological needs like experience feelings of competence, social relatedness with others, or feeling self-determined in their everyday life (e.g., in their professional life) are not satisfied.

### Basic Psychological Needs Thwarting and the Compensation Effect

The self-determination theory provides a general framework on psychological functioning and how it affects individuals’ effort, agency, and commitment in life. According to Deci and Ryan’s (1985, 2000) self-determination theory, three innate psychological nutriments are essential for the individuals’ psychological, social, and physical well-being. These are the basic psychological needs for competence, autonomy, and social relatedness. The need for *competence* is the desire to experience feelings of competence and the mastery of challenging tasks (e.g., Deci and Ryan, 2000; see also White, 1959). Need for *autonomy* is the constant desire to experience volition control and self-ownership (deCharms, 1968). The need for *social relatedness* is the attempt to feel socially connected to other people (Baumeister and Leary, 1995; Deci and Ryan, 2000).

Several studies in different domains of life have confirmed the theoretical assumption that the satisfaction of basic psychological needs (e.g., through environments that support those needs) has positive consequences. Basic need satisfaction, for example, leads to emotional well-being (Parfitt et al., 2000; Edmunds et al., 2007; Aldrup et al., 2017) and motivation (Wilson et al., 2007; Devloo et al., 2015) in sports (further examples of the application of basic need theory in sports: Mullan et al., 1997; Pelletier and Sarrazin, 2007; Vallerand, 2007; Lundqvist and Raglin, 2015; see Hagger and Chatzisarantis, 2007, for a summary).

Most importantly for the present research question, Deci and Ryan (2000; see also Ryan et al., 1995) assume that when basic needs are not fulfilled, people may try to cope in maladaptive ways. Two examples of this are need substitutes and rigid behavior (Deci and Ryan, 2000). Regarding the former, individuals with low need satisfaction develop need *substitutes* (prestigious objects, social approval), which might improve their subjective well-being in the short run but fail to contribute to their well-being in the long run (which is only achieved through stronger basic need satisfaction) (e.g., Vansteenkiste et al., 2007; Soenens et al., 2008). *Rigid behavior* is defined as avoidance of changes in behavioral contingencies according to one’s own values (Hayes et al., 1999). Such rigid behavior like exaggerated perfectionism in one’s professional life, might also be beneficial in the short run, because it provides structure and is associated with predictability (Shafran and Mansell, 2001). However, it also fails to satisfy basic psychological needs and consequently is an important predictor of ill-being. One example of a rigid pattern of behavior as a response to impaired need satisfaction is restricted eating. Eating disorders represent a comorbidity (Szabo et al., 2018) of exercise dependence and the relationship between unfulfilled needs and problems associated with restricted eating is empirically supported (Thogersen-Ntoumani and Ntoumanis, 2007; Stok et al., 2010; Froreich et al., 2017). Summarizing the main assumptions of this research field, people often try to compensate for low basic need satisfaction in important life domains by trying to reach higher need satisfaction in other life domains. In the worst case, this can be done through restricted eating, for example, or – as we suggest in our present research – by exercising excessively. Restricted eating might be a person’s attempt to compensate for low *autonomy* satisfaction (Stok et al., 2010). For example, individuals try to gain control over their hunger in order to experience autonomy (which they do not experience in other life domains). We assume that exercising excessively also results from basic need thwarting. This should be especially true for basic need for competence satisfaction. We hypothesize that a highly achievement-oriented setting such as extreme endurance sport is a person’s attempt to compensate for low basic need for *competence* satisfaction in other life domains (e.g., at the work place, in leisure time). For example, individuals try to improve their running time and enjoy training progress in order to overcome need for competence frustration in their professional lives. Similar theoretical considerations might have caused Lalande et al. (2017) to derive their hypotheses. They suggest that two distinct sources of need satisfaction have to be distinguished. Need satisfaction inside and outside the activity. In accordance with their hypotheses the authors found that need satisfaction inside an activity (doing sports, doing music) leads to harmonious passion (a balanced type of passion associated with positive consequences for motivation and well-being), whereas low need satisfaction outside the activity leads to a compensatory striving which results in an unhealthy type of passion in the activity called obsessive passion (for details about the differentiation of obsessive and harmonious passion see Vallerand, 2008). Although the studies conducted by Lalande et al. (2017) encourage our line of reasoning outlined above, they differ with regard to the central concept (passion rather than exercise dependence) and with regard to the range of analyzed activities [broad range in ((s))Lalande et al. (2017) study, among them: music, basketball, arts and crafts, reading, work]. In the present research we focus on the domain of sport and, most importantly, we analyze extreme behavior (extreme endurance sport).

Furthermore, rather than combining the items of the three needs into an overall measure of need satisfaction (see Lalande et al., 2017), we aim to disentangle the effects of the three needs. As introduced above, we assume that the basic need for competence plays a crucial role in the achievement-oriented activity (extreme endurance sport) which we analyzed in our study. As a further theoretical extension of previous research, we aim to shed light on the mechanism that links low basic need satisfaction with exercise dependence.

### Anxious Self-Motivation as a Mediator in the Basic Need Exercise Dependence Relationship

As already stated above, we assume that low basic need for competence satisfaction outside exercising might be an antecedent of exercise dependence. However, in contrast to previous studies on the effects of need frustration that mainly focussed on facets of well-being (e.g., well-being, vitality; see above), the core of exercise dependence is the (maladaptive) persistence in *behavior.* Although exercise addiction is accompanied by impairment of well-being, its core feature is excessive behavior. Therefore, we assume that a motivational variable is necessary to link the frustration of felt competence, relatedness and autonomy with the initiation and maintenance of excessive behavior. We assume that this variable is *anxious self-motivation* (Kuhl, 2000) due to the following theoretical considerations. According to ((s))Kuhl (2000) theory of volition, low basic need satisfaction is a state of stress and frustration, which is assumed to be related to reduced utilization of volitional functions. Among the consequences of volitional inhibition, such as *energy deficit* and *heightened proneness to external control* (which might elicit passivity, but not active coping), Kuhl and Fuhrmann (1998, p. 29) suggest “a tendency to engage in negative motivation control in terms of anticipating negative consequences of not acting or not reaching the goal.” In their *Volitional Component Inventory* (short form; German version), the authors call this facet of self-control *anxious self-motivation*, meaning that fear motivates the investment of time and effort in order to prevent failure or further frustration and stress. A similar anxiety-driven mechanism has been proposed in sport, for example by Conroy and Coatsworth (2007) who empirically confirmed that basic need frustration is responsible for the fear of failure of youth swimmers. Hall et al. (2007) further showed that maladaptive forms of achievement orientation (e.g., overstriving, neurotic perfectionism) are predictors of obligatory exercise.

Concisely stated, basic need frustration leads to an avoidance-oriented form of self-control which aims to avoid negative states (e.g., low need satisfaction), but activates rather than inhibits behavior (engagement, excessive behavior rather than passivity). In statistical terms and referred to the domain of extreme endurance sport, we assumed that anxious self-motivation mediates the relationship between athletes’ basic need for competence frustration and exercise dependence.

These considerations are also in agreement with SDT theorizing (Ryan and Deci, 2000) which suggests that when basic needs are thwarted, behavior is guided by less autonomous and hence maladaptive forms of regulation (e.g., avoidance rather than approach regulation). Like other forms of avoidance motivation (see Elliot, 2008), anxious self-motivation in turn leads to behavior aimed at avoiding undesired states. These undesired states may be experienced in the work domain or the person’s private life. As already stated above, anxious self-motivation may then guide a person’s behavior toward strong involvement in excessive compensatory exercise, to avoid feelings of reduced competence.

Summing up we derived the following hypotheses from our reasoning outlined above. Our first hypothesis is that low need satisfaction in one’s professional and private life may lead to excessive involvement in the domain of endurance sports, in which feelings of competence may seem to be relatively easy to attain (progress and success mainly depend on oneself and are easy to measure). Our second hypothesis concerning the mechanism is that low need satisfaction activates anxious self-motivation, which in turn results in an dependence to the exercise activity. Furthermore, we assume that Hypotheses 1 and 2 are especially true for the basic need for competence due to the performance-oriented nature of extreme endurance sports.

## Materials and Methods

### Participants and Procedure

We recruited a sample of 323 ultra-endurance athletes (269 men) with a mean age of 45.72 years (*SD* = 9.20) via the mailing lists of ultra-endurance events and through personal contacts. We selected a sample of ultra-endurance athletes who take part in competitions exceeding 6 h in duration. Forty-nine participants were multiple triathletes, 131 described themselves as long-distance runners, 14 as long-distance swimmers, and 91 as long-distance cyclists. Thirty-eight participants chose the response “other kinds of endurance sports.” The inclusion criterion was that they had participated in an ultra-endurance event (defined as a competition that exceeds 6 h in duration). Because we did not find differences between the different types of sport, we used the entire sample for the analyses reported below.

Participants filled in an anonymous web survey (LimeSurvey) including questions on their age, sex, and their main sports. Furthermore, it contained the basic psychological need satisfaction measures for professional and private life, and for sports. In order to separate the two basic need measures from another, we used 16 filler items, which are unrelated to the present research question (FKS, Rheinberg et al., 2003). Furthermore, the web survey contains the self-control measure, and the exercise dependence measure.

### Measures

In order to measure the *basic psychological needs* for competence, social relatedness and autonomy satisfaction in professional and private life and in sports, we used a German translation of the Balanced Measure of Psychological Needs Scale (BMPN; Sheldon and Hilpert, 2012; German translation by the first author). Each scale consists of six items (e.g., competence*: “I was successful at completing difficult tasks and projects*,” social relatedness: “*I felt in touch with people who care for me, and whom I care for,”* autonomy*: “I was free to do things my own way”*). Participants were first asked to relate the statements to their extreme endurance sport activity and then (after the filler items) to their professional and private life outside sports. Instructions to the second questionnaire were that we (the experimenters) are well aware that filling in the same questionnaire twice might be a bit boring or annoyingly, but that we ask them to fill the questionnaire thoroughly and with regard to the domains of work and other leisure time activities (expect sports). They rated their agreement with the statements using a 7-point rating scale ranging from 1 (strongly disagree) to 7 (strongly agree). We computed a basic need for competence satisfaction, need for relatedness satisfaction and need for autonomy satisfaction score as well as an overall need satisfaction score (mean of the three subscales), separately for the domains sport and work/private life. Whereas the Cronbach’s Alphas for need satisfaction at work/private life were sufficiently high (competence: 0.72; relatedness: 0.69; autonomy: 0.79; overall score: 0.84), the Alphas were lower for basic need satisfaction in sports (competence: 0.50; relatedness: 0.55; autonomy: 0.61; overall score: 0.72).

In order to assess anxious self-motivation, we used the *anxiety-oriented self-control* (named *goal orientation without anxiety* in the original scale; *reversed* items) subscale of the Volitional Components Inventory (German version: Selbststeuerungsinventar, SSI-K3; Kuhl and Fuhrmann, 1998; Kuhl and Alsleben, 2009). Participants used a 4-point rating scale (1: strongly disagree – 4: strongly agree) to indicate their agreement with statements such as “*In order to motivate myself, I often imagine what would happen if I didn’t finish the task on time*.” The means of participant’s responses to the four items were used as their score for anxious self-motivation (Cronbach’s Alpha = 0.63).

*Exercise dependence* was assessed using the 6-item Exercise Addiction Inventory (Terry et al., 2004). It consists of components of behavioral dependence suggested by Griffith (1996) (see above) (e.g., salience: “*Exercise is the most important thing in my life*”; tolerance: “*Over time I have increased the amount of exercise I do in a day*”; withdrawal symptoms: “*If I have to miss an exercise session, I feel moody and irritable*”). Participants indicated their agreement with the statements using a 5-point rating scale ranging from 1 (strongly disagree) to 5 (strongly agree). The overall score for exercise addiction (mean of all items) was sufficiently reliable, with Cronbach’s alpha = 0.72. At a cut-off score of 24 (top 15%) athletes are considered at-risk of exercise addiction (Terry et al., 2004).

## Results

### Preliminary Analyses, Descriptive Statistics and Correlations

Preliminary analyses showed that the participants’ age did not influence the results reported below. Men and women did not differ in any of the assessed variables and the participants’ gender did not influence the reported results.

**Table 1** shows the means and standard deviations of the relevant variables and their inter-correlations. As can be seen, the basic need scores in sports and at the workplace/leisure time are highly correlated with each other (*r*’s between 0.17 and 0.84, all *p*’s < 0.01). Furthermore, basic needs for competence in both domains are significantly negatively correlated with anxious self-motivation (*r* = –0.21 for sport and *r* = –0.20 for work/leisure time). Differences between the sport and the work/leisure domains were found in the relationship with exercise addiction. As predicted, only basic need for social relatedness (*r* = –0.16, *p* < 0.01), need for autonomy (*r* = –0.11, *p* < 0.01), and the overall need satisfaction score (*r* = –0.15, *p* < 0.01) for work/leisure were negatively related with exercise addition. An exception was basic need for competence satisfaction which was unrelated to exercise addiction (*r* = –0.07, *p* = 0.27). In contrast, the scores of need satisfaction in sports were unrelated to exercise addiction (all *r*’s < 0.10).

**Table 1:** Descriptive statistics and correlations (Pearson, two-tailed, *N* = 323).

	1	2	3	4	5	6	7	8	9	10	*M*	*SD*
1 Competence_Sport	-	0.41^∗∗∗^	0.37^∗∗∗^	0.78^∗∗∗^	0.52^∗∗∗^	0.23^∗∗∗^	0.18^∗∗^	0.38^∗∗∗^	-0.21^∗∗∗^	-0.06	5.60	0.79
2 Relatedness_Sport		–	0.26^∗∗∗^	0.78^∗∗∗^	0.33^∗∗∗^	0.46^∗∗∗^	0.20^∗∗^	0.41^∗∗∗^	0.05	-0.08	5.42	0.91
3 Autonomy_Sport			–	0.67^∗∗∗^	0.24^∗∗∗^	0.26^∗∗∗^	0.28^∗∗∗^	0.33^∗∗∗^	-0.01	-0.01	5.68	0.90
4 NeedSat_Sport				–	0.49^∗∗∗^	0.41^∗∗∗^	0.29^∗∗∗^	0.49^∗∗∗^	-0.06	-0.06	5.54	0.64
5 Competence_Work					–	0.36^∗∗∗^	0.50^∗∗∗^	0.78^∗∗∗^	-0.20^∗∗∗^	-0.07	5.47	0.96
6 Relatedness_Work						–	0.41^∗∗∗^	0.74^∗∗∗^	-0.09	-0.16^∗∗^	5.47	0.96
7 Autonomy_Work							–	0.84^∗∗∗^	-0.04	-0.12^∗^	4.90	1.18
8 NeedSat_Work								–	-0.13^∗^	-0.15^∗∗^	4.28	0.82
9 Anx.SelfMot									–	0.16^∗∗^	1.98	0.70
10 Exercise dependence											4.07	1.15


*^∗^p < 0.05. ^∗∗^p < 0.01. ^∗∗∗^p < 0.001. NeedSat_Sport/Work: overall score for basic need satisfaction. Anx.SelfMot: anxious self-motivation.*

### Test of the Mediation Model

In order to examine whether anxious self-motivation mediates the relationship between basic psychological need satisfaction and exercise dependence, we used Hayes (2013) PROCESS macro (Model 4) to perform a bootstrapping procedure (5000 bootstraps) and a Sobel test. We repeated this analysis separately for the three basic needs and the overall score for both life domains (sport and work/leisure) and summarized the results in **Table 2**. The indirect effects for basic need for competence satisfaction, but not for the needs for relatedness and autonomy were significant. Furthermore, the overall score of need satisfaction for the work/leisure domain was marginally significant (and most likely due to the need for competence score).

**Table 2:** Indirect effects of the mediation analyses using normal distribution (Sobel test) and bootstrapping for basic psychological need satisfaction (competence, relatedness, autonomy, overall score) in the sport and work/leisure domains as the predictors, anxious self-motivation as the mediator and exercise dependence as the dependent variable.

	Indirect effects		Sobel tests		95% CI (Bootstrap)	
	*b*	*seb*	*z*	*p*	*LL*	*UL*
Competence_Sport	-0.05	0.02	-2.14	0.03	-0.092	-0.009
Relatedness_Sport	0.01	0.01	0.77	0.44	-0.012	0.037
Autonomy_Sport	-0.002	0.01	-0.19	0.48	-0.029	0.024
NeedSat_Sport	-0.01	0.02	-0.87	0.38	-0.051	0.017
Competence_Work/Leisure	-0.03	0.02	-2.05	0.04	-0.073	-0.007
Relatedness_Work/Leisure	0.01	0.01	-1.29	0.20	-0.045	0.005
Autonomy_Work/Leisure	-0.01	0.01	-0.67	0.50	-0.026	0.010
NeedSat_Work/Leisure	-0.03	0.01	-1.65	0.09	-0.063	-0.001


*NeedSat_Sport/Work: overall score for basic need satisfaction.*

**FIGURE 1:**
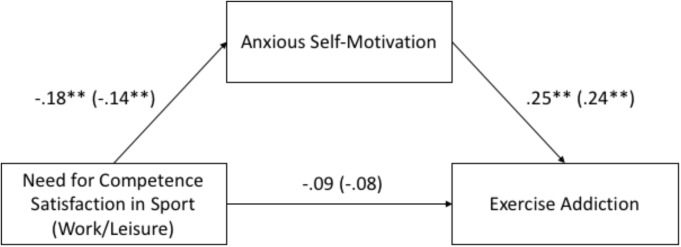
Illustration of the significant mediation models. Need for competence satisfaction in the sport and in the work/leisure (coefficients in brackets) domain predicts exercise addiction through anxious self-motivation. ^∗∗^*p* < 0.01.

The nature of the significant mediation analyses for basic need for competence satisfaction are displayed in **Figure 1**.

## Discussion

The present study supported our hypothesis and the underlying compensation hypothesis that low basic need for autonomy and social relatedness satisfaction outside the sport activity (work/leisure time) is associated with exercise addiction, whereas the same need satisfaction scores in sport are unrelated. An exception is the basic need for competence in the work/leisure domain, which was unexpectedly not directly linked to exercise addiction. Its relation to exercise addiction can only be found once anxious self-motivation is considered as a mediator. Low basic need for competence satisfaction, for example at work, makes people anxiously self-motivated. Driven by fear and the attempt to avoid further unfulfilled needs they engage excessively in behavior that promises competence experience (e.g., exercising). A set of mediation analyses might have supported our assumptions about highlighting the need for competence satisfaction (rather than relatedness or autonomy for which no significant mediation effects could be revealed). The competence satisfaction → anxious self-motivation → exercise addiction mediation also holds true for need satisfaction experienced in the activity (exercising) itself. It seems plausible that a lack of competence feelings in endurance sports motivates people to invest more effort into the activity; particularly because effort investment is usually a successful strategy in this kind of sport. However, an unhealthy type of motivation results, when anxious self-motivation lies at the heart of this attempt.

Future research is needed to address several open research questions linked to our assumptions and findings, such as whether the athletes are consciously aware that they compensate frustrated basic needs in one domain (e.g., at the work place) by engaging in alternative activities (exercising). As already stated, the compensatory approach is not limited to the need frustration-compensatory behavior pair that we analyzed in the present study. The compensatory approach could also mean that people aim to compensate their need frustration in sports with excessive work (workaholism) or with drinking alcohol (when drinking alcohol makes them feel competent). Thus, it could be of interest to evaluate other types of behavioral addictions and substance addictions as potential need compensatory activities (Taylor and Thompson, 2018). A further question could address what distinguishes individuals who respond with exercise dependence or other types of dependence from those who do not (test of a moderated mediation).

Through our research, we have added to the existing literature on exercise dependence by combining motivational and self-control antecedents as explanations for exercise dependence: Whereas previous research on exercise dependence has focussed on physiological reasons for exercise addiction, such as the release of endorphins, which are responsible for euphoric feelings during exercise (Morgan, 1985), and on learning processes such as the positive consequences of exercising (relaxation, mastery experience, goal attainment), which reward the activity and enhance the likelihood of performing it again, we have added low basic psychological need satisfaction and anxious self-motivation to the list of variables which lead to exercise dependence. As mentioned before, the present study proposes that basic psychological need of competence thwarting in other life domains may lead to excessive sport behavior especially when athletes feel afraid of further need thwarting. For this reason they might excessively participate in sports because this provides controllable satisfaction of the need for competence.

Our study also contributes to basic psychological need theory. The few studies that have linked self-determination theory with maladaptive exercise behavior, such as exercise dependence (Frederick and Ryan, 1993; Hamer et al., 2002), have focussed on the mode of self-regulation (ranging from external, introjected, identified, integrated to intrinsic regulation; see Deci and Ryan, 1985) in sport rather than assuming a compensatory mechanism, as in the present study. These studies found that introjected and identified regulation (already relatively self-determined forms of regulation) in sports are positive predictors of exercise dependence. This indicates that a strong engagement in exercise requires some degree of self-determination (Hall et al., 2007).

One study which considered the level of basic need satisfaction in addition to forms of self-control was conducted by Edmunds et al. (2005). However, the authors relate need satisfaction to exercise activity (e.g., “*I feel like I am free to decide for myself how to exercise*”; “*Most days I feel a sense of accomplishment from exercising*.”; “*People I exercise with take my feelings into consideration*”) rather than to other life domains in which basic need satisfaction might be low (as we did in assuming a compensatory mechanism). They found that athletes displaying symptoms of dependence reported higher levels of need for competence (but not need for autonomy and relatedness) compared with individuals who do not exhibit symptoms of dependence. Furthermore, they displayed higher levels of identified, integrated and intrinsic motivation. This supports the above-mentioned assumption that a high level of commitment in sports, or even exercise dependence, requires a certain degree of self-determination.

It must also be pointed out here that the ultra-endurance athletes in our study score relatively high in the exercise addiction measure with a mean score right at the cut-off point (24 – 4.0 at item level, see **Table 1**). This means we could investigate the relationship between need satisfaction, avoidance motivation, and exercise dependence in a high-risk sample for whom these results are very important.

Although the present study mainly supports our hypothesis, it is important to point out that our results must be interpreted with caution due to relatively weak effects (e.g., correlations between variables were significant, but low) and a lack of replication so far. However, our approach has practical implications that differ from the implications derived from previous explanatory models. It suggests that a more profound way of addressing critical exercise behavior (than for example, by reducing the amount of exercise behavior using techniques of impulse control) would be to improve need satisfaction in other life domains and in particular basic need for competence satisfaction. If people feel competent at their workplace, for example, they will not need compensatory behavior that endangers their health. Examples of how competence satisfaction can be fostered are regular feedback and well-structured contexts, structure in the form of guidelines, rules and norms which are not experienced as being controlling. These are essential in order to feel competent and autonomous (Vansteenkiste et al., 2010, p. 123).

Another methodological limitation of the present study is that it is based on correlational data. Causal interpretations are thus not warranted. Long-term studies which examine the association between the two variables over an extended period of time (for example, from beginning with an endurance sport up to an extreme endurance sport level) could answer the question of causality more appropriately. We speculate that such a study would reveal a circular interplay between basic need satisfaction and exercise addiction in the sense that need frustration leads to exercise dependence, which in turn aggravates conflicts in one’s social and professional life and further leads to low basic need satisfaction.

We would like to end this report with the statement that it is important to analyze the conditions under which sport and exercise become harmful. We fully agree with the current research trend positing that exercise is physically and psychologically beneficial to health and that it is absolutely necessary to examine, support and create the conditions under which individuals exercise regularly. However, we would also like to suggest that it is important to analyze the conditions under which sport and exercise become harmful. Previous research and our own study results indicate that (the presence or absence of) basic psychological needs are a core concept for explaining the health-promoting initiation and maintenance of exercise behavior, as well as health-endangering excessive exercise behavior. In this regard, the present article additionally suggests that not only basic needs – described as innate psychological “nutriments” (Ryan, 1995, p. 410) – but also self-control processes (anxious self-motivation) co-determine whether or not individuals are at risk from exercise dependence.

## Ethics Statement

This study was carried out in accordance with the recommendations of the University of Zürich, Ethics committee of the Institute of Psychology. The protocol was approved by the Ethics committee of the Institute of Psychology. All subjects gave written informed consent in accordance with the Declaration of Helsinki.

## Author Contributions

JS and BK gathered the data. JS, BK, and MW wrote the article. JS performed the statistical analyses.

## Conflict of Interest Statement

The authors declare that the research was conducted in the absence of any commercial or financial relationships that could be construed as a potential conflict of interest.
